# Case report: The use of metagenomic next-generation sequencing to diagnose Lemierre’s syndrome caused by lost root canal fillings in a 33-year-old patient with metabolic syndrome

**DOI:** 10.3389/fmed.2024.1402444

**Published:** 2024-08-14

**Authors:** Yimeng Zhou, Yongzhen Zhai, Yan Wang, Dan Zhang, Guohe Feng

**Affiliations:** Department of Infectious Diseases, Shengjing Hospital of China Medical University, Shenyang, China

**Keywords:** Lemierre’s syndrome, *Fusobacterium necrophorum*, mNGS, metabolic syndrome, case report, odontogenic infection

## Abstract

**Background:**

Lemierre’s syndrome is a rare and serious complication of pharyngitis with an estimated annual incidence of 1 in 100,000 people worldwide. It is characterized by septic thrombophlebitis of the internal jugular vein with metastatic infection, usually after oropharyngeal infection. Rare cases of Lemierre’s syndrome have been reported to be caused by odontogenic infection.

**Case report:**

A 33-year-old male visited our hospital with symptoms of fever and sore throat for 16 days. The other symptoms included pain in his left neck and shoulder. In addition, metabolic syndrome was diagnosed based on waist circumference, diabetes, and hyperlipidemia. *Fusobacterium necrophorum* bacteria was detected using the metagenomic next-generation sequencing (mNGS) technique. The enhanced computerized tomography (CT) scan showed thrombosis of the left proximal jugular vein and brachiocephalic vein. Based on these observations, Lemierre’s syndrome was diagnosed. The etiology was that the fillings in the root canal tooth were lost with no blood or pain about 2 weeks before the onset. The patient recovered after treatment with antibiotics and blood purification.

**Conclusion:**

Lemierre’s syndrome should be evaluated for patients with fever, sore throat, and neck pain. If the loss of fillings from root canal therapy occurs, especially for those with metabolic syndrome, we should be aware of the possibility of this disease. Furthermore, the mNGS test can be used as a crucial supplementary diagnostic tool for patients with undetermined fever.

## 1 Introduction

Lemierre’s syndrome was first proposed and named by French physician Andrè Lemierre in 1936. The disease was mainly manifested as an oropharyngeal infection, accompanied by thrombosis of the internal jugular vein and metastatic infection (1). With the use of antibiotics, the incidence of Lemierre’s syndrome, known as the “forgotten” disease, has declined (2). However, perhaps due to the decline in the empirical use of antibiotics for oropharyngeal infections, the number of cases has increased worldwide since the late 1970s, and it remains a rare disease with a high fatality rate of 5–18% (3). Most cases occur in healthy young people aged 15–30 years and initially manifest as pharyngeal infections, such as pharyngitis and tonsillitis, while some cases occur among elderly people who have underlying diseases and manifest as distant infections, such as empyema or brain abscess (4). The odontogenic infections account for only 1% of overall cases (5). The current case is a young patient with Lemierre’s syndrome accompanied by metabolic syndrome, due to the loss of fillings from root canal therapy in the left maxillary first premolar.

## 2 Case report

A 33-year-old male patient, height 175 cm, weight 84 kg, waist circumference 102 cm, and a smoking history for 10 years, with no history of previous illness and drinking came to our hospital on 28 August 2021, with a main complaint of “fever, sore throat for 16 days, diarrhea for 1 day.” The patient had developed a fever and sore throat 16 days ago, and the temperature decreased after the administration of oral roxithromycin. He felt pain in his left neck and shoulder 12 days ago. There was tenderness on the left side of the neck and palpable swollen lymph nodes when the patient was examined in the clinic. He was given intravenous quinolones for their broad antimicrobial coverage. On that day, the patient passed stool more than 10 times and went to the fever clinic of our hospital. The physical examination revealed a heart rate of 88 beats/min, blood pressure of 80/50 mmHg, respiration of 18 beats/min, clear consciousness, and moderate yellowing of the skin. There was no enlargement of the tonsils, no abnormality of the heart and lungs, mild tenderness under the xiphoid process, and no palpation of the liver and spleen. Initial laboratory results are given in Table 1. Chest computerized tomography (CT) revealed multiple nodules and cavitary lesions in both lungs. Abdominal CT showed the proximal jejunum wall was slightly thickened with exudation and fatty liver. The diagnosis was “septic shock; pulmonary infection; liver damage; acute renal failure; metabolic acidosis; ion disorder; fatty liver,” and the patient was admitted to the hospital for treatment. The bacterial blood culture and the metagenomic next-generation sequencing (mNGS) were conducted to detect the bacterial pathogen. The treatments included administering sodium bicarbonate to correct acidosis, fluid complement, imipenem for treating severe infection, etc.

**Table 1:** Laboratory results of the patient on admission.

Indices	Results	Normal values
*Gas analysis*		
pH	7.33	7.35–7.45
PaO_2_ (mmHg)	101	75–100
PaCO_2_ (mmHg)	16.9	35–45
HCO_3_^−^ (mmol/L)	8.7	22–26
Extracellular base excess (mmol/L)	−16.3	−3 to 3
SO_2_ (%)	96.5	95–100
Blood lactic acid (mmol/L)	3.02	0.7–2.1
*Hematological test*		
White blood cell count (×10^9^/L)	14.6	3.5–9.5
Neutrophils (%)	85.9	43.2–71.5
Hemoglobin (g/L)	116	130–172
Platelet count (×10^9^/L)	9	135–350
*Biochemical and immunological test*		
Total protein (g/L)	47.9	63–80
Albumin (g/L)	17.6	35–53
Alanine aminotransferase (U/L)	79	0–40
Aspartate aminotransferase (U/L)	99	5–34
Total bilirubin (μmol/L)	107.7	3.4–20.5
Conjugated bilirubin (μmol/L)	90.1	0–8.6
Blood urea nitrogen (mmol/L)	33.92	3–9.2
Creatine (μmol/L)	446	59–104
Creatine kinase (U/L)	45	29–200
Creatine kinase MB (U/L)	12.5	0–24
Troponin I (μg/L)	0.4186	0–0.0198
Myohemoglobin (μg/L)	142.8	0–105.7
Blood glucose (mmol/L)	9.09	3.9–6.11
Serum potassium (mmol/L)	3.23	3.5–5.5
Serum sodium (mmol/L)	122.1	136–145
Serum chlorine (mmol/L)	88.2	96–108
C-reactive protein (mg/L)	230.1	0–8
Procalcitonin (ng/mL)	>100	<0.05
*Coagulation functions*		
Prothrombin time (s)	15.6	9.4–12.5
Activated partial thromboplastin time (s)	37.8	21–37
Fibrinogen (g/L)	4.66	2–4
D-dimer (μg/L)	2,726	0–252

**Figure 1:**
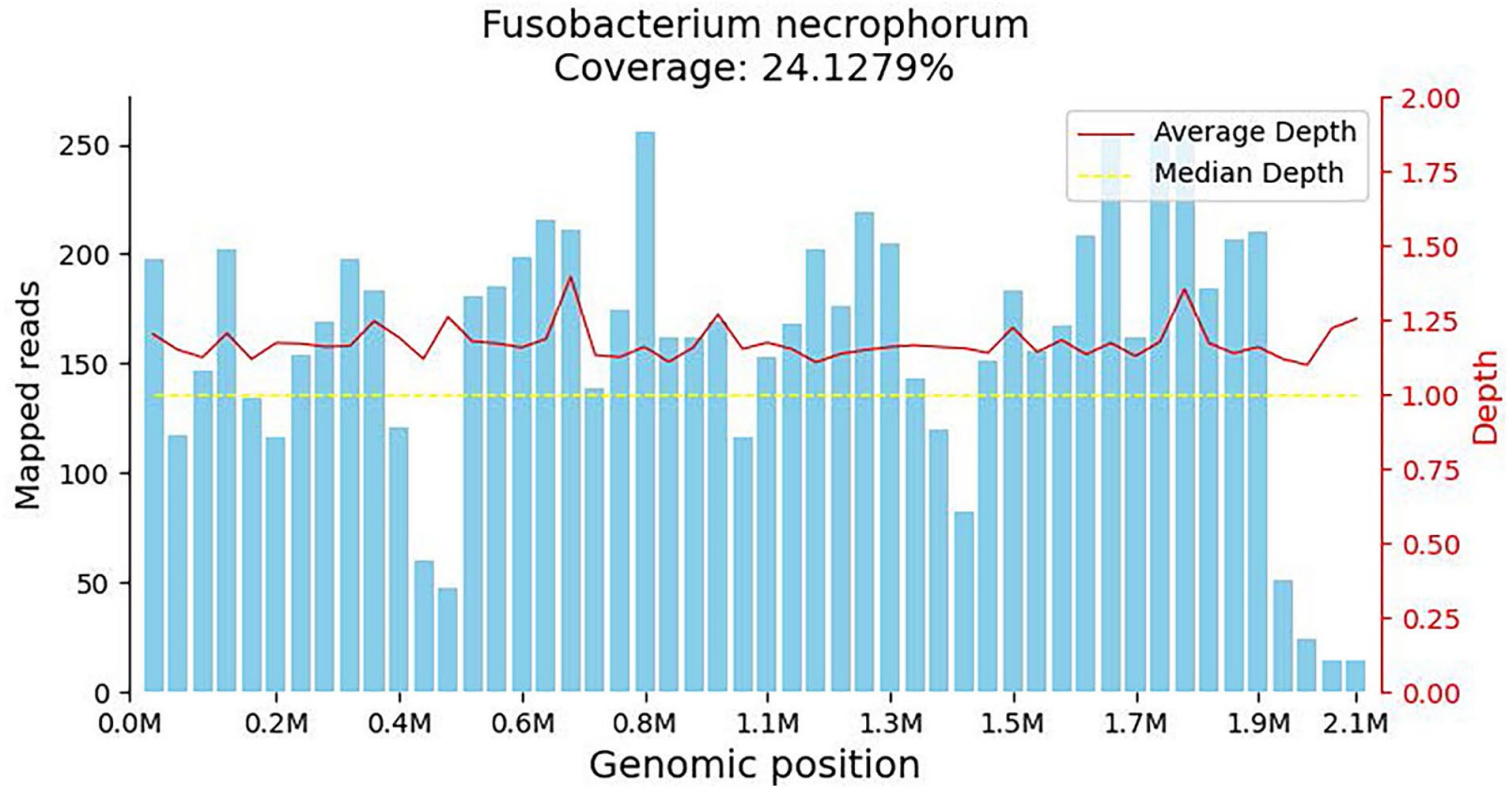
Coverage map for *Fusobacterium necrophorum*, reflecting the distribution of sequences aligned to the genome of the microorganism. The horizontal axis represents the size of the microorganism’s genome, while the vertical axis represents the number of sequences detected within different genomic regions.

**Figure 2:**
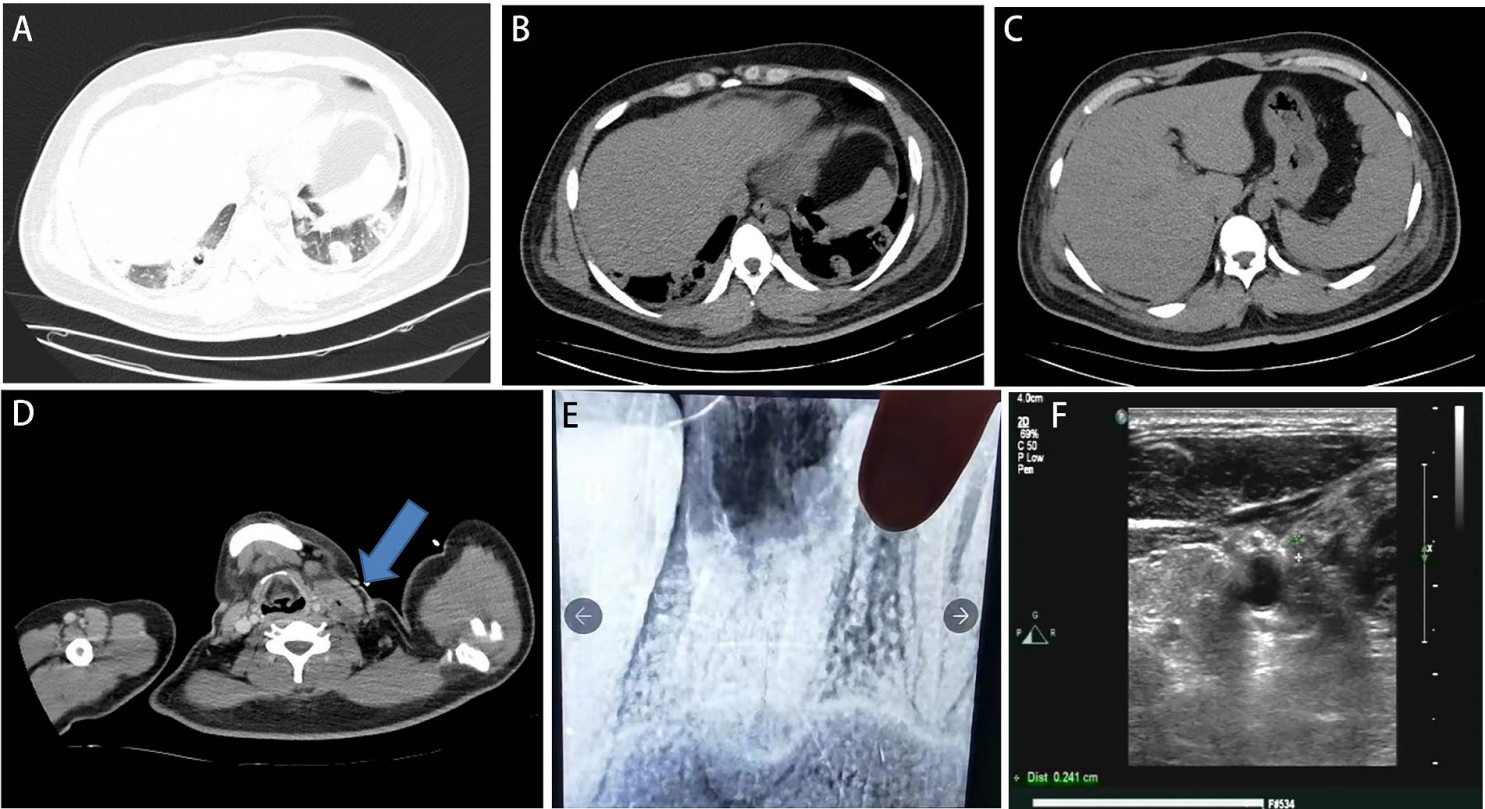
**(A–D)** Computed tomography scan at admission to the intensive care unit. **(A,B)** It showed bilateral pulmonary nodules and cavities. **(C)** Decreased liver density. **(D)** Left internal jugular vein thrombosis with gas accumulation (blue arrow). **(E)** X-ray after charge in a dental clinic. A cavity was at the left maxillary first premolar. **(F)** 2 months after discharge, the ultrasound showed that the thrombus in the neck had not subsided.

The patient developed agitation, gibberish, and emotional agitation and was transferred to the intensive care unit the next day. Since increased bilirubin combined with consciousness was observed, thrombotic thrombocytopenic purpura (TTP) cannot be excluded. ADAMTS13 activity was detected, and plasma exchange was conducted for three consecutive days. Meropenem was given due to its ability to penetrate the blood–brain barrier. The laboratory tests showed triglyceride of 4.06 mmol/L, high-density lipoprotein cholesterol (HDL-C) of 0.07 mmol/L, glycosylated hemoglobin of 8.7%, fasting blood glucose of 10.51 mmol/L, suggesting diabetes and hyperlipidemia. On the 5th day of admission, the bacterial blood culture showed anaerobic Gram-negative bacilli. However, the specific pathogen and drug susceptibility tests were not conducted. The mNGS test showed *Fusobacterium necrophorum* (Identification confidence 99%, 13,723 reads; genus *Fusobacterium*, relative abundance 85.6%, and 14,370 reads) (Figure 1). The patient was conscious and complained of pain in the left neck and shoulder and limited activities. The enhanced CT scan showed that the left proximal jugular vein and brachiocephalic vein had a strip filling defect, indicating thrombosis. The surrounding soft tissue was swollen, with a small amount of gas accumulation, multiple nodules, and cavitary lesions in both lungs increased accompanied by the consolidation of some lung tissues (Figures 2A–D). Based on these indications, Lemierre’s syndrome was diagnosed. Nadroparin calcium was administered as an anticoagulant. ADAMTS13 activity showed 26%, excluding TTP. The infectious indicators of the patient decreased, while the liver and kidney function improved. On the 9th day of admission, the patient was transferred to the infectious disease ward. Rivaroxaban was given as an anticoagulant. On the 18th day of admission, the antibiotic was changed to cefoperazone/sulbactam sodium 3 days before discharge. After repeated medical history queries, the patient mentioned he underwent root canal treatment on the left maxillary first premolar 20 years ago, and the fillings in the tooth fell out about 2 weeks before onset. Rivaroxaban was continued after discharge. Then he took an oral X-ray at the dentist’s clinic (Figure 2E). The chest CT scan was found to be normal when taken 2 months after discharge. However, the ultrasound showed that the thrombus in the neck had not subsided (Figure 2F). The anticoagulant drugs were discontinued.

## 3 Discussion

The lung is the most common site of septic embolism in Lemierre’s syndrome. The other pus emboli dissemination sites include joints, soft tissues, abdominal parenchyma, central nervous system, etc. (6). When the patient came to our hospital, his symptoms were already 16 days old, with fever and diarrhea as the main manifestations. The procalcitonin and C-reactive protein were significantly increased, and the chest CT scan showed multiple nodular cavitation infections. We considered it a pulmonary infection caused by the spread of severe sepsis in the blood. The patient had left neck and shoulder pains in the early stage, but no relevant examinations were performed. After admission, the pain with limited mobility did not attract our attention. When the mNGS test showed the presence of *F. necrophorum*, it was concluded to be thrombophlebitis caused by this bacterium, leading to disseminated lung infection. Common signs and symptoms of internal jugular vein thrombosis include pain, swelling, or induration along with the sternocleidomastoid muscle on the ipsilateral side of the neck and mandible, with high fever (7). When the abnormal symptoms and signs of the neck appear at the beginning, an ultrasound or CT examination was conducted to detect a thrombus, and thus, Lemierre’s syndrome was diagnosed early. Fortunately, the mNGS result provides us with a rapid diagnosis.

mNGS is a rapid and efficient sequencing method with a shorter detection timeframe compared to traditional culture methods. It excels in identifying pathogens responsible for sepsis more effectively than other techniques. Interpreting mNGS results involves comprehensive analysis based on factors such as sequence count, coverage, pathogen abundance, and supporting evidence. Despite its higher cost, mNGS offers a distinct advantage in diagnosing uncommon pathogens during critical infections (8). The clinical presentation of Lemierre’s syndrome at admission was atypical, and blood cultures only yielded anaerobic organisms without specific species or drug susceptibilities. Fortunately, the mNGS test showed that the patient was infected with *F. necrophorum*, providing clues to the diagnosis of Lemierre’s syndrome. The patient received timely and appropriate antibiotic treatment.

The median age for the onset of Lemierre’s syndrome is 21 years (9). This age distribution is unclear and may be related to various factors. Host factors include tonsil surgery, frenulum surgery, combined Epstein–Barr virus infection, immune status, hormone use, blood hypercoagulability, etc. (10). In most cases, the primary infection is associated with pharyngitis of the palatine tonsils or peritonsillar tissue, and odontogenic infections are less common. After primary infection, local invasion of the pharyngeal space and internal jugular vein causes septic thrombophlebitis at intervals of 1–3 weeks (11). In a retrospective analysis of 99 cases of Lemierre’s syndrome from 2001 to 2008, only seven cases (7.1%) had an odontogenic infection as the primary source of infection (12). We searched “PubMed” for the last 20 years, and 24 case reports confirmed Lemierre’s syndrome was caused by odontogenic infections (Table 2). Among them, periodontal disease was the main cause in 54.17% (13/24), including symptoms of periodontitis, gingivitis, and dental caries, followed by surgical operation in 33.33% (8/24). Our case denied previous periodontal disease and recent surgeries. Root canal treatment was performed 20 years ago. The filler from the root canal treatment fell out 2 weeks before the onset, and there was no discomfort such as bleeding or swelling. This etiology has not been reported in previous cases.

**Table 2:** Literature review of reported Lemierre’s syndrome caused by odontogenic infection.

Investigator	Dental origin	Source pathogens	Age	History
Klinge (13)	Periodontal disease	*Fusobacteria*	12	Healthy
Tan (12)	Dental caries, periodontitis	*Fusobacterium necrophorum*	78	Diabetes mellitus
Sonsale (12)	Dental abscess	*Fusobacterium necrophorum*	8	Healthy
Shibasaki Warabi (12)	Dental caries	Negative	59	Glaucoma, hyperlipidemia
Juárez Escalona (12)	Periodontal disease, dental caries	*Streptococcus intermedius*; *Bacteroides fragilis*	36	Healthy
Duquesne (12)	Gingivitis	*Fusobacterium necrophorum*	11-month	Healthy
Malis (14)	Tooth extraction	*Alpha-hemolytic Streptococcus*	23	Healthy
Rosado (12)	Dental caries	*Streptococcus salivarius*	38	Healthy
Gupta (15)	A dental scraping of molars	*Streptococcus intermedius*	Middle-aged	Epilepsy
Wu (16)	Toothpick usage	*Fusobacterium necrophorum*	17	Healthy
Ghaly (17)	Placement of a prosthesis	Negative	44	Diabetes mellitus
Oya (18)	Cut the gum	*Fusobacterium necrophorum*	33	Healthy
Noy (14)	Dental caries	*Staphylococcus capitis* *alpha-hemolytic streptococci*	30	Healthy
Miyamoto (19)	Gingivitis, periodontitis, tooth extraction	Negative	66	Pulmonary tuberculosis at around 40 years
Chua (20)	A cavity at the molar tooth	*Klebsiella pneumoniae*	53	Diabetes mellitus
Scopel Costa (21)	Periodontitis	Negative	34	Hypertension, obesity
Li (22)	Odontogenic infections	*Prevotella buccae*; *Streptococcus anginosus*; *Streptococcus constellatus*	79	Diabetes mellitus
B (23)	Dental caries	*Streptococcus Intermedius*	37	Epilepsy
Clark (24)	Tooth extraction	Negative	24	Splenectomy; pyruvate kinase deficiency
Caruso (25)	Tooth extraction	*Streptococcus intermedius*	65	Hypertension, depression
Halawa (26)	Tooth extraction	*Streptococcus anginosus*	61	Healthy
Zhang (27)	Periodontal disease	*Fusobacterium necrophorum*	35	Healthy
Tran (28)	Tooth extraction	*Streptococcus anginosus*	56	Hypertension, diabetes mellitus, dyslipidaemia
Santos (29)	Tooth extraction	Negative	31	Healthy

*Fusobacterium necrophorum* is an anaerobic Gram-negative bacillus in the normal flora of the oral pharynx, intestine, and female reproductive tract, and is the most common pathogen causing Lemierre’s syndrome. It can cause various pathogenic factors, such as endotoxin, leukotoxin, proteolytic enzymes, hemagglutinin, and hemolysin. It has strong endotoxin characteristics because its lipopolysaccharide accounts for 4% of the cell wall content (30). The patient’s platelets were as low as 7 × 10^9^/L due to hemagglutinin, leading to platelet aggregation (31). Pathogens were recognized in 18 of 24 patients reviewed in the literature, *F. necrophorum* in 8 (44.44%) and *Streptococcus intermedius* in 4 (22.22%) patients. There were 12 patients with underlying diseases; among them, eight patients had symptoms of metabolic syndrome such as hypertension, diabetes, hyperlipidemia, and obesity. The infection in our patient was caused by *F. necrophorum*. Diabetes, hyperlipidemia, and fatty liver were diagnosed based on laboratory tests, and metabolic syndrome was diagnosed based on waist circumference. In addition, smoking is a major risk factor for cardiovascular disease. The patient’s history of smoking is a predisposing factor for his metabolic syndrome and increases his risk of infection (32). Although no relationship between metabolic syndrome and Lemierre’s syndrome of odontogenic infection has been reported, metabolic syndrome is likely a predisposing factor. Periodontal disease is a risk factor for metabolic syndrome (33). In a Korean analysis of periodontal disease status and metabolic syndrome among adults, periodontitis severity was positively associated with hypertriglyceridemia and low HDL-C in men, low HDL-C and abdominal obesity in women, and positively related to metabolic syndrome (34). In a study in 2015, 8 of 9 patients with *Klebsiella* pneumonia-associated Lemierre syndrome had poorly controlled diabetes (35). In addition, periodontal disease was significantly associated with non-alcoholic fatty liver disease (36). Potential liver damage from periodontal disease may be hematogenously transmitted to the liver and may contribute to liver disease progression (37). The root canal fillings fell out about 2 weeks before the onset of symptoms. Although there were no clear symptoms indicating that the periodontal tissue was damaged, the metabolic syndrome indicated the mucosal barrier function could have been broken. The onset of symptoms also conforms to the time of post-infection cervical phlebitis, causing the invasion of *F. necrophorum*.

*Fusobacterium necrophorum* is generally sensitive to penicillin, clindamycin, metronidazole, and chloramphenicol, and responds differently to second- and third-generation cephalosporins. A retrospective analysis of 96 patients found that carbapenems and piperacillin/tazobactam are commonly used with/without metronidazole for Lemierre’s syndrome, and 98% of cases were successfully treated (38). We diagnosed our patient for septic shock caused by Gram-negative bacilli and chose imipenem for treatment. After neurological symptoms, we switched to meropenem, which can penetrate the blood–brain barrier. Since TTP could not be excluded, plasma exchange was selected to remove inflammatory mediators. The duration of the blood culture test for anaerobic bacteria is long. After timely treatment, the patient’s indicators for infection decreased significantly, and he returned to normal temperature.

At present, there is a controversy about the anticoagulant treatment of the disease. A recent retrospective study of the efficacy of anticoagulation in 51 patients with Lemierre’s syndrome with jugular vein thrombosis found no significant difference in outcomes among patients with jugular vein thrombosis who received therapeutic, prophylactic, or no anticoagulation treatment. Two patients who did not receive anticoagulants were diagnosed with septic arthritis during hospitalization, and one patient developed major bleeding while receiving a therapeutic dose of anticoagulant (39). The patient in this report was treated with nadroparin calcium initially and then changed to oral rivaroxaban. After 3 months of medication course, the thrombus still existed, and rivaroxaban was discontinued. The patient’s neck thrombosis still needed to be followed up. The ultrasound should be reviewed every 3 months, and if there is no change in the thrombosis or any clinical symptoms, no treatment is needed. However, if there is a new thrombus or with symptoms, such as neck swelling and pain persist, endovascular treatment will be needed.

## 4 Conclusion

We reported a young case of Lemierre’s syndrome caused by odontogenic infection based on a metabolic syndrome. The reason is the loss of fillings from root canal therapy in the molar teeth. Early symptoms of Lemierre’s syndrome can be subtle, and abnormal symptoms and signs in the neck are easily overlooked. Therefore, for patients with fever, sore throat, and neck pain, when the mentioned odontogenic etiology is noted, especially those with metabolic syndrome, we should be aware of the possibility of this disease. In addition, the mNGS can be used as a crucial supplementary diagnostic tool for patients with undetermined fever. To obtain promising results, timely and individualized treatment of anticoagulant drugs, antibiotics, and blood purification should be performed with respect to the patient’s conditions.

## Data availability statement

The raw data supporting the conclusions of this article will be made available by the authors, without undue reservation.

## Ethics statement

The studies involving humans were approved by the Ethics Committee of Shengjing Hospital of China Medical University (No. 2022PS651K). The studies were conducted in accordance with the local legislation and institutional requirements. Written informed consent for participation was not required from the participants or the participants’ legal guardians/next of kin in accordance with the national legislation and institutional requirements. Written informed consent was obtained from the participant/patient(s) for the publication of this case report.

## Author contributions

YiZ: Writing – original draft, Resources, Methodology, Conceptualization. YoZ: Writing – review & editing, Formal analysis. YW: Writing – review & editing, Investigation. DZ: Writing – review & editing, Data curation. GF: Writing – review & editing, Visualization, Validation, Supervision.
